# Putting patients first: how to carry out a patient-centred eye examination

**Published:** 2019-12-17

**Authors:** Renee du Toit, Elmien Wolvaardt

**Affiliations:** 1 Consultant, Pretoria, South Africa.; 2 Editor: *Community Eye Health Journal*, International Centre for Eye Health, UK.

**We can provide better care if we focus on our patients as human beings, not just on their eyes.** ^1^

**Figure:**
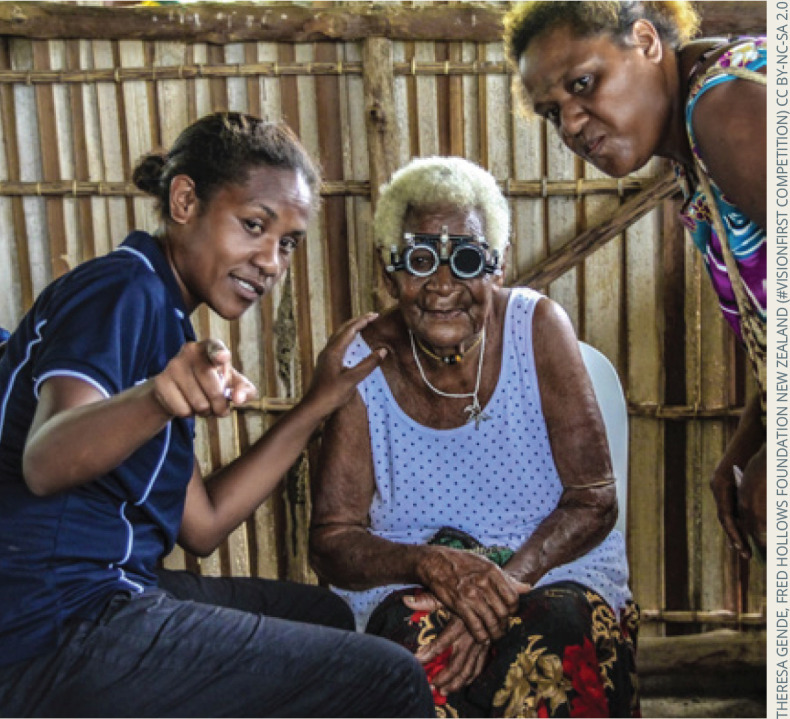
Explaining visual acuity testing to a patient. PAPUA NEW GUINEA

**Before carrying out any eye procedure**

Wash your hands (and afterwards too)

Use gloves if indicated (e.g., for an invasive procedure or if an eye appears infectious)

Clean or disinfect equipment

Ensure that lighting is appropriate

Clearly explain what you are going to do

Position the patient comfortably.

1. **Consider the person as a whole**
  From when you first meet your patients, notice how they use their vision. Are they able to walk around by themselves? Is there any evidence of pain? What other health conditions or disabilities do they have?
2. **Establish a good relationship**
  Greet the person warmly. Introduce yourself by name and explain your role in everyday language, e.g.: “I am here to look at your eye(s) so we can find out what is wrong and how to help you.” Speak in a respectful, kind and compassionate manner, and take time to get to know the person as an individual.
3. **Listen**
  When you are taking a history, it is very important to listen very carefully; do not interrupt the patient or jump to conclusions. Ask how symptoms affect patients' daily living, and whether they have any concerns or fears, such as a fear of blindness or having eye surgery. Find out what their expectations are about the outcomes of treatment.
4. **Make the patient comfortable and tell them what you are doing**
  Before measuring visual acuity or carrying out an examination, tell patients what you will be doing and explain what you would like them to do, e.g., point in the direction the of the letters on a tumbling E chart. If it is a longer procedure, talk them through the steps, particularly if they cannot see what you are doing. Ensure patients are positioned comfortably and encourage them to tell you if they experience pain or discomfort. Some patients may not feel able to tell you, so check their face periodically for any visible signs of pain and make adjustments as needed.
5. **Talk about what comes next**
  Explain whether any further tests are necessary, or whether a referral to a specialist is needed. Tell patients what treatment they might require, including where to get medicine and how to use it. Ask them if they have any questions and ensure that they and their carers/relatives (if appropriate) have all the information they need, such as the address and clinic times if they are referred.
